# Evaluation of Different Lactic Acid Bacteria as Starter Cultures for Nono—A West African Fermented Dairy Product

**DOI:** 10.3390/foods13193030

**Published:** 2024-09-24

**Authors:** Onyeka M. Ikele, Chigoziri T. Ogu, Xiuping Jiang, George A. Cavender

**Affiliations:** 1 Department of Applied Microbiology and Brewing, Nnamdi Azikiwe University, Awka 420110, Nigeria; oikele@clemson.edu (O.M.I.); ct.ogu@unizik.edu.ng (C.T.O.); 2 Department of Food, Nutrition and Packaging Sciences, Clemson University, Clemson, SC 29634, USA; xiuping@clemson.edu

**Keywords:** cultured dairy, nono, sensory evaluation, starter culture, lactic acid bacteria, fermented milk

## Abstract

Nono is a traditional cultured dairy product consumed across West Africa. In this study, five cultures isolated from Nigerian-produced nono and three purified lactic acid bacteria from the USDA-NRRL were examined for use in preparing nono starter cultures. Isolated cultures were characterized using microbiological and biochemical tests, including 16s rDNA sequencing to identify the genotype. Each isolated strain was cultured and inoculated into UHT milk (1% *v*/*v*) and allowed to ferment for 24 h at 25 °C. Fermented products were evaluated for pH, moisture content, water activity, and viscosity, and their descriptive sensory properties were noted. The isolate that resulted in sensory properties most similar to traditional nono was then used as the primary strain for subsequent starter culture blends made with the NRRL cultures. These blends were used for the fermentation of nono and compared with commercial nono samples. Isolates obtained from nono were as follows: *Lactobacillus fermentum*, *Lactobacillus paracasei*, and, surprisingly, *Lactobacillus rhamnosus*, which has not been previously reported as a part of the nono microflora. There was no significant difference in the physical parameters of nono made from the individual indigenous isolates and a similar pattern was observed for the organisms from NRRL, except that their total titratable acidity and viscosities were significantly (*p* < 0.05) higher than those of the indigenous organisms. Compounded starter made with *L. rhamnosus* and NRRL cultures was then used to make nono that showed significantly (*p* < 0.05) different pH and viscosity values than commercially purchased nono, while sensory evaluation showed that nono made from the new starter culture had a high overall consumer acceptance score.

## 1. Introduction

Nono is a fermented dairy product made in different parts of Nigeria and West Africa, especially in settlements inhabited by the Fulani tribe. Nono production is known to be a craft and also a source of livelihood for the Fulani women who hand-milk the cows and ferment the milk into nono; they are popularly called ‘milkmaids’. Nono holds a cultural heritage for the Fulani tribe, who are known to be traditionally pastoral nomadic farmers who reside in different parts of West Africa [1,2,3]. The culture of nomadic pastoralism made it possible for the consumption of nono to spread from the Fulanis to other tribes in the region where they reside at a given time. On the other hand, nomadic pastoralism poses a disadvantage to the safety and quality of nono made by these women, since there are no standard fermentation facilities available and no standardized product-processing methods. Other disadvantages are product inconsistencies between the different nono batches made, as well as the presence of microbial contaminants and pathogens in the finished product.

Nono is essentially made through hand milking, overnight boiling of the milk, cooling and fermentation, storage, and vending. As much as the boiling step is a critical control point in its production process, contamination occurs at the fermentation step, stemming from the process of back-slopping with improperly preserved cream (pre-ferment) from the previous fermentation batch [2]. This cream (*Manshanu*) is purported to be the starter culture for the fresh fermentation process; however, it is usually preserved in a wooden calabash kept in a hut. The cream has been found to be heavily laden with pathogens and fecal contaminants [3,4,5,6,7,8], evidently due to the association of nono with houseflies. This poses a food safety risk t consumers; therefore, methods to provide a safe and wholesome product are paramount. Likewise, variations in product consistency abound as a result of differences in the microbial composition of each cream used for back-slopping.

Nono has been reported to be a beverage rich in carbohydrates, proteins, and minerals [2,9,10]. It contains free fatty acids, lactose, calcium, sodium, potassium, magnesium, iron, and zinc [2,5]. It is also known to be rich in probiotic microorganisms, especially those of the lactic acid bacteria group. Nono is produced through lactic acid fermentation, and different studies carried out on nono have shown the presence of lactic acid bacteria, viz., *Lactobacillus brevis*, *Lactobacillus casei*, *Lactobacillus fermentum*, *Lactobacillus plantarum*, *Lactobacillus bulgaricus*, *Lactobacillus senioris*, *Lactobacillus helveticus*, and *Streptococcus thermophilus*, *Lactocossus lactis*, *Leuconostoc pseudomesenteroides*, and *Lactobacillus cremoris* [8,11,12,13,14,15,16,17].

However, these listed microorganisms do not all appear in one product; instead, they occur in variations in the different nono products examined. These organisms are known to drive the fermentation process, impacting the physical and sensory properties of nono—alongside the possible metabolic contributions of contaminants and pathogens like *Staphylococcus aureus*, *Alcaligenes faecalis*, *Clostridium sporogenes*, *Salmonella*, and *Escherichia coli* [18,19,20]. Thus, this study sought to evaluate different lactic acid bacteria reported to be indigenous to nono, for the sole purpose of creating a beneficial consortium that serves as the best starter culture with positive consumer acceptance and, by extension, solves the food safety problem.

## 2. Materials and Methods

### 2.1. Materials

Shelf-stable UHT milk (Horizon Organic, USA) was sourced from a local supermarket. Microbial media (MRS agar and MRS broth) were sourced from VWR Avantor, Radnor, PA, USA. pH buffers, indicators, and chemicals (ethanol and sodium hydroxide) were also sourced from VWR Avantor, USA.

### 2.2. Isolation, Characterization, and Identification of Indigenous Lactic Acid Bacteria in Nono

Ten nono samples were pooled and subjected to 1 in 10-fold serial dilution in phosphate-buffered saline (pH 6.8). Subsequently, 0.1 mL of the 10^−3^ tube from each sample was cultured on MRS agar plates and incubated at 30 °C and 5% CO_2_ in an anaerobic incubator for 24–48 h. This was carried out according to the modified methods of Fagbemigun [20]. Isolates obtained from the cultured plates were separated into pure cultures based on their colony morphologies and thereafter subjected to a Gram stain, catalase test, and oxidase test for preliminary identification.

Subsequently, 16s rDNA sequencing was used to identify the isolates to the species level at Zymobiomics, Orange, CA, USA. The DNA samples were prepared for targeted sequencing with the Quick-16S ŒPlus NGS Library Prep Kit (Zymo Research, Irvine, CA, USA). These primers were custom-designed by Zymo Research to provide the best coverage of the 16S gene while maintaining high sensitivity, and the actual sequencing was performed by the aforementioned commercial lab (Zymobiomics, Irvine, CA, USA) as part of their commercial offerings.

Three lactic acid bacterial samples—*Lactobacillus plantarum*, *Lactobacillus casei,* and *Lactococcus lactis*—from fermented milk were also obtained from the USDA-NRRL culture collection and used as extraneous isolates for the product formulation.

### 2.3. Nono Production

#### 2.3.1. Standardization of Starter Culture Isolates

The method reported by Ikele et al. [21] was used to standardize the starter cultures used for the fermentation process. Briefly, a 0.25 mL aliquot of pure culture isolates (10^5^ cfu/mL) of each lactic acid bacterium was incubated in 25 mL of MRS broth without agitation at 30 °C for 24 h and then used as an inoculum to begin the fermentation process.

#### 2.3.2. Fermentation Protocol

A modified method described by Adesokan [12] was used for nono production. For the lab fermentation procedure, 1% (*v*/*v*) of each indigenous isolate in De Man, Rogosa, and Sharpe (MRS) broth was inoculated into UHT-pasteurized whole milk (Grade A organic, Horizon Organic, Broomfield, CO, USA) in situ, sealed, and incubated at 30 °C for 24 h.

All production trials for analytical/instrumental analyses were performed in triplicate.

### 2.4. Physiochemical Analysis

Determinations of pH, moisture content, water activity, viscosity, total titratable acidity, and color were performed on the samples of nono. The pH was determined using a digital pH meter (pHenomenal, VWR, Randor, PA, USA). The pH electrode was immersed in 10 mL of the sample until a stable reading was obtained, and the values were recorded.

To determine moisture content, the samples were placed dropwise onto an aluminum pan provided by the manufacturer of a halogen moisture analyzer (model number 677723 Schuler Scientific, Englewood, CA, USA) and allowed to run to completion using the built-in sensing feature. Water activity determination was likewise carried out using a specialized instrument, in this case an Aqualab water activity meter (model number 1100843 Aqualab, Pullman, WA, USA), with the samples being loaded into disposable sample cups before initiating the measurement cycle.

Viscosity was measured using a rotary viscometer (model number 126408 Produstrial, Fredon, NJ, USA). For each measurement, the viscometer probe was immersed in a 15 mL aliquot of the sample before rotation was initiated, and the sample was allowed to reach a stable reading before the values were recorded.

Total titratable acidity determination was carried out according to the titration method described by Fabro [22]. Briefly, a 20 mL aliquot of fermented milk samples was added to 40 mL of distilled water that had been boiled and cooled, along with 2 mL of phenolphthalein solution as an indicator (prepared by dissolving 1% phenolphthalein in 95% ethanol). The mixture was then titrated with 0.1 M NaOH until a pink color change was observed.

To determine instrumental color, a 10 mL aliquot of each fermented milk sample was dispensed into the lid of individual 100 mm Petri dishes and covered with the inverted dish body, which was then placed onto the white calibration tile provided by the colorimeter manufacturer. A calibrated handheld colorimeter (model number CR400, Konica Minolta, Ramsey, NJ, USA) was then used to determine the color through the Petri dish.

#### Preliminary Sensory Property Screening

Test nono samples made from each of the indigenous isolates were examined for characteristic appearance, taste, aroma, and texture by the research team. Isolates which exhibited characteristic sensory properties were selected as choice isolates for the starter culture formulation study.

### 2.5. Evaluation of Effects of Best Starter Culture Consortium on Physical and Sensory Properties of Nono

The choice isolate from the indigenous cultures was used in bi- and multiple-culture cocktails with the extraneous fermented milk cultures from the USDA-NRRL at 1% (*v*/*v*), at 30 °C for 24 h, for the fermentation process. The physical and sensory parameters of the fermented products were measured as previously described. Unfermented fresh milk and a commercially purchased nono were used as the control for this experiment. Sensory evaluation of the finished product was carried out by ten untrained panelists (who are frequent nono consumers) from Nnamdi Azikiwe University, Awka, Nigeria, using a 9-point hedonic test according to the modified methods of Dafur [19]. They characterized the nono on attributes of appearance, taste, aroma, and texture.

Institutional oversight was provided by Nnamdi Azikiwe University, under their existing approval for food tastings. Participant consent was obtained via oral consent in accordance with the published oral consent guidelines provided by the Department of Applied Microbiology and Brewing, Nnamdi Azikiwe University, Awka, Nigeria. Panelists who did not provide consent or withdrew consent were excluded from this study and any data regarding their responses were destroyed.

### 2.6. Statistical Analyses

The data obtained were analyzed as means with analyses of variance (ANOVA) using statistical software (GraphPad Prism, version 10.3.1). Results were deemed significant if *p* ≤ 0.05.

## 3. Results

### 3.1. Isolation, Characterization, and Identification of Indigenous Lactic Acid Bacteria in Nono

**Figure 1:**
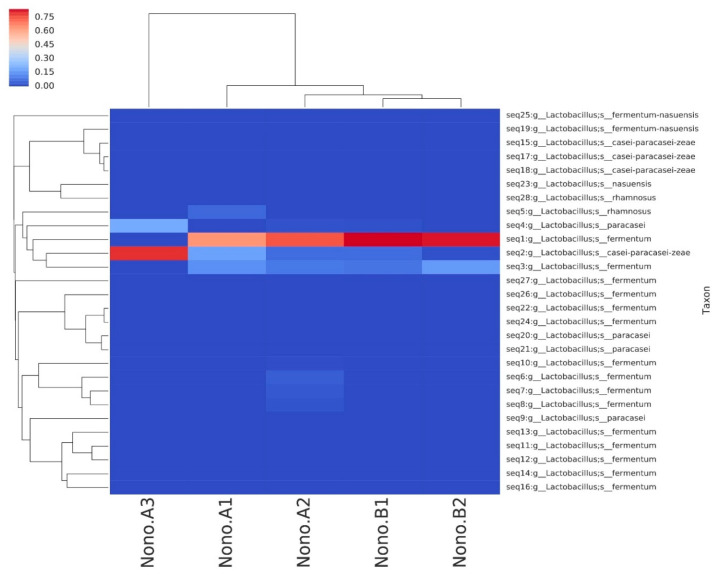
16s rDNA amplicon mapping of isolates.

Five *Lactobacillus* isolates (A–E) were isolated from the examined nono samples on the basis of their colony morphologies, and their biochemical characteristics are shown in Table 1. Molecular characterization of the isolates through sequence blast identified them as *Lactobacillus fermentum* (three isolates), *Lactobacillus paracasei,* and *Lactobacillus rhamnosus* (Table 1); a presumptive microbial identity heat map is also shown in Figure 1.

### 3.2. Assessment of Effects of Starter Culture Isolates on Physical and Sensory Properties of Nono

**Figure 2:**
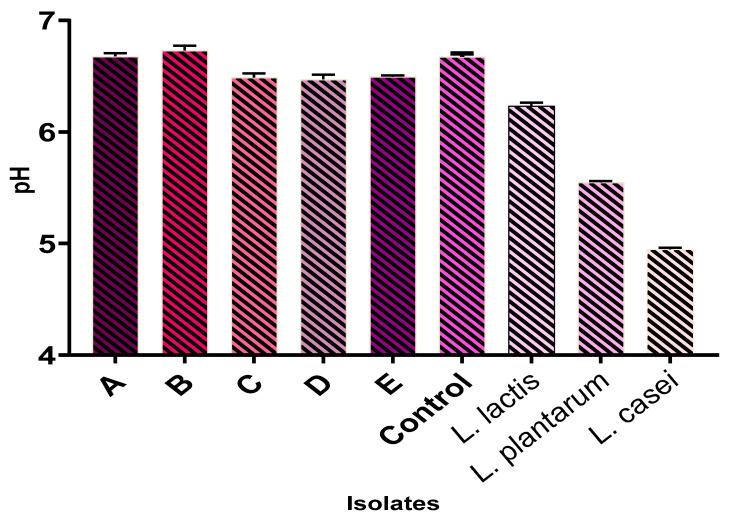
pH values of each starter culture candidate after fermentation. A: *Lactobacillus fermentum* 1; B: *Lactobacillus paracasei*; C: *Lactobacillus fermentum* 2; D: *Lactobacillus fermentum* 3; E: *Lactobacillus rhamnosus*.

**Figure 3:**
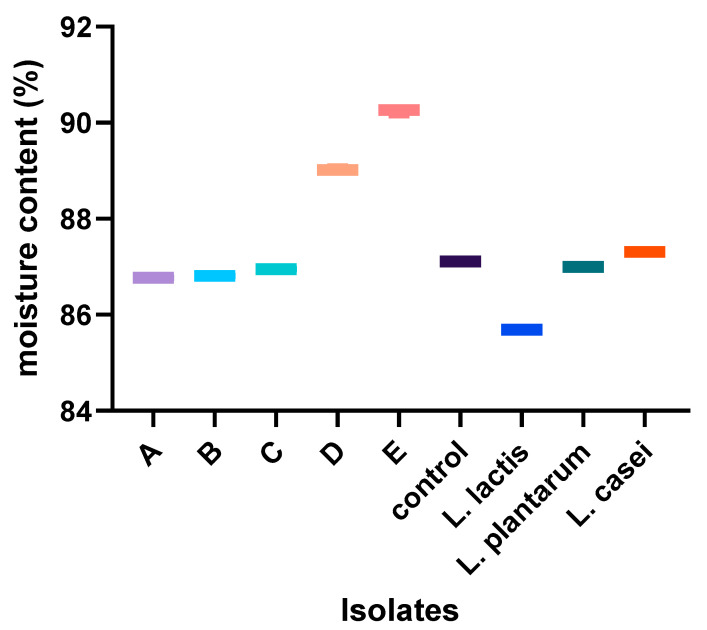
Moisture content values of each starter culture candidate after fermentation. A: *Lactobacillus fermentum* 1; B: *Lactobacillus paracasei*; C: *Lactobacillus fermentum* 2; D: *Lactobacillus fermentum* 3; E: *Lactobacillus rhamnosus*.

**Figure 4:**
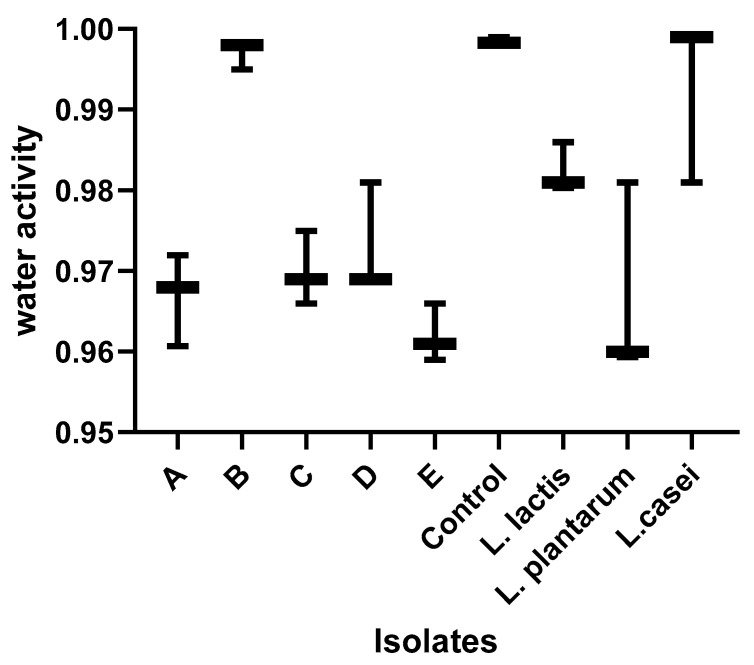
Water activity values of each starter culture candidate after fermentation. A: *Lactobacillus fermentum* 1; B: *Lactobacillus paracasei*; C: *Lactobacillus fermentum* 2; D: *Lactobacillus fermentum* 3; E: *Lactobacillus rhamnosus*.

**Figure 5:**
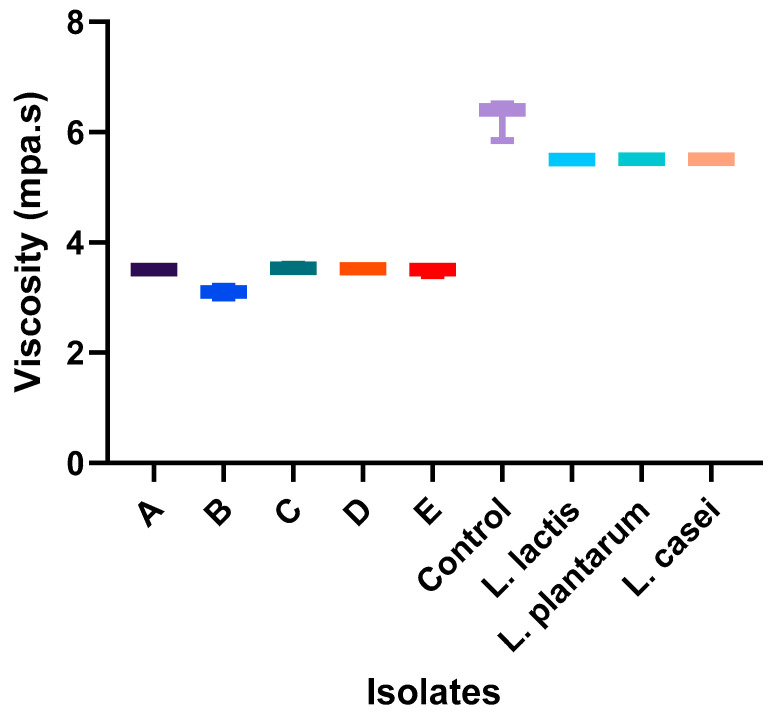
Viscosity values of each starter culture candidate after fermentation. A: *Lactobacillus fermentum* 1; B: *Lactobacillus paracasei*; C: *Lactobacillus fermentum* 2; D: *Lactobacillus fermentum* 3; E: *Lactobacillus rhamnosus*.

**Figure 6:**
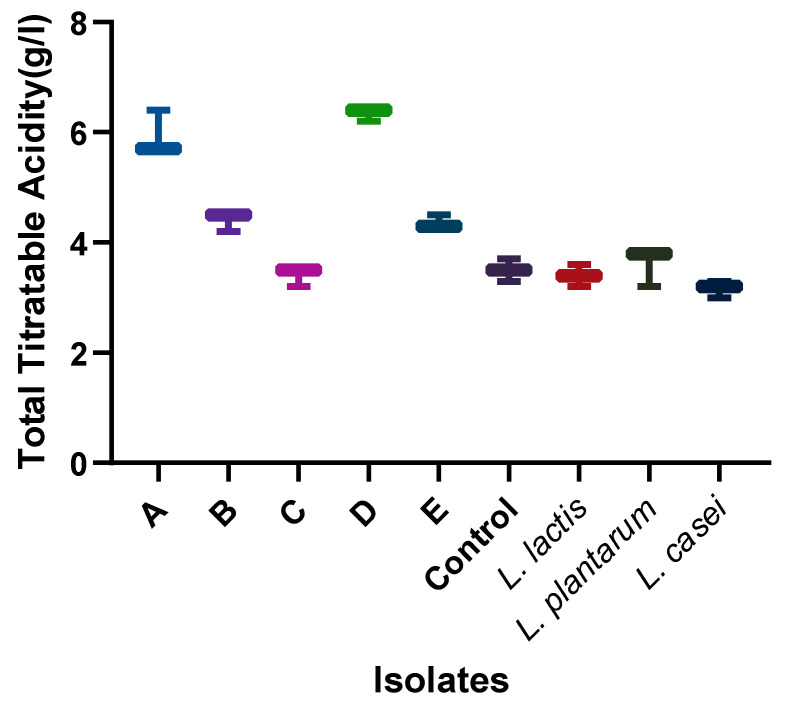
Total titratable acidity of each starter culture candidate after fermentation. A: *Lactobacillus fermentum* 1; B: *Lactobacillus paracasei*; C: *Lactobacillus fermentum* 2; D: *Lactobacillus fermentum* 3; E: *Lactobacillus rhamnosus*.

**Figure 7:**
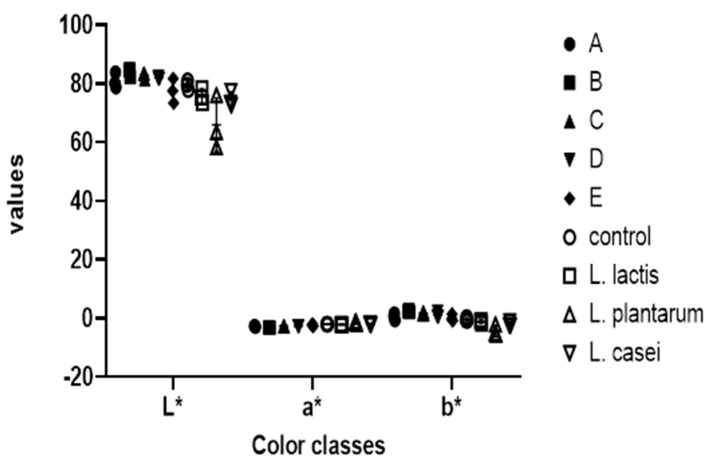
Color values of each starter culture candidate after fermentation. A: *Lactobacillus fermentum* 1; B: *Lactobacillus paracasei*; C: *Lactobacillus fermentum* 2; D: *Lactobacillus fermentum* 3; E: *Lactobacillus rhamnosus*.

Indigenous starter culture isolates exhibited a low acid pH with no significant (*p* > 0.05) difference between cultures. For most of the isolates, no difference was found in moisture content (except for *L. rhamnosus*), water activity (except for *L. paracasei*), color, and viscosity values compared with those of the nono fermented with each indigenous isolate. For the extraneous starter cultures, there were no significant differences in the pH (except for *L. casei*), moisture content, and color (except for *L. plantarum*) when compared to the indigenous cultures. However, nono produced from these extraneous cultures had significantly (*p* < 0.05) higher total titratable acidity (3.0–3.8 g/L) and viscosity values (5.5 mpa.s) when compared to the indigenous cultures, as shown in Figure 2, Figure 3, Figure 4, Figure 5, Figure 6 and Figure 7. Screening of the sensory capacities of indigenous and extraneous isolates is shown in Table 2.

### 3.3. Evaluation of Effects of Best Starter Culture Consortium on Physical and Sensory Properties of Nono

*Lactobacillus rhamnosus* exhibited the best sensory quality in the fermented product that typified that of the regular nono and was made the choice isolate. This isolate was then used in co-culture with the extraneous isolates as bi-cultures and mixed cultures. The mixed culture displayed the physical properties (Table 3) and sensory properties (Table 4) most consistent with conventional nono.

## 4. Discussion

The present study sought to formulate a starter culture suitable for nono production with the goal of its standardization and improved safety. The lactic acid cultures isolated and used in this study have been partly reported by [12,15,20] as part of the microflora of nono. However, this study identified *Lactobacillus rhamnosus*, which has not been previously reported by different authors [8,11,12,13,14,15,16,17] as part of the microflora of nono. This study isolated *Lactobacillus fermentum*, *Lactobacillus paracasei*, and *Lactobacillus rhamnosus*. *L. fermentum* was the most commonly occurring isolate, which corresponds with the reports of [15,20], who also reported *L. fermentum* in nono. Three milk-fermenting lactic acid cultures not isolated from this study, but reported to be a part of the nono microflora [12,20], were also obtained from the USDA-NRRL culture collection center and incorporated as a part of the starter culture consortium in order to conduct a fair assessment on the fermentation capacities of each microbe reported to be found in nono.

The suitability of these organisms as starter culture candidates was assessed in terms of the physical and sensory properties they imparted on the fermented product, while Adesokan [12] chose the suitability of their own isolates for starter culture formulation solely on the basis of diacetyl production capacity. For the indigenous isolates used in this study, there was no significant (*p* > 0.05) difference observed in the pH, moisture content, and viscosity when they were used individually as starter cultures. The pH values of nono made from each isolate ranged from 6.4 to 6.7, which partly corresponds with the reports of Adisokan et al. [12] and Dafur et al. [19] and differs entirely from the reports of Abdulrahman et al. [1], Omola et al. [2], and those of Nebedum and Obiakor [9]. Adesokan [12] also reported a moisture content range of 80.7- 87.11 for nono made from their starter culture experiment, which corresponds partly with that of this study. When compared to the fresh whole milk used for the control experiment, these indigenous isolates impacted the product and significantly (*p* < 0.05) increased viscosity and total titratable acidity in the fermented milk. According to Bachmann [23], viscosity is a key physical property that affects the textural property of fermented milk products, stemming from the microbial binding of water through exopolysaccharide formation during the fermentation process. Obioha [15,24] reported that these microbial exopolysaccharides produced during fermentation increase water retention in the fermented milk product/nono; this may explain the results of the present study, which showed no significant (*p* > 0.05) difference in the moisture content values of the test nono products and the unfermented milk sample. Of the extraneous organisms used in the starter culture, *Lactobacillus casei* and *L. plantarum* showed decreased pH values (4.95 and 5.46, respectively), but they were not statistically significant (*p* < 0.05) compared to the pH values of other starter culture candidates. Li [25] reported that lactic acid bacteria are known for medium acidification and the coagulation of milk during fermentation. These extraneous cultures (*L. lactis*, *L. plantarum,* and *L. casei*) produced a significantly (*p* < 0.05) viscous product compared to that of the indigenous cultures when used singly as starter cultures. Therefore, they were used in the starter culture consortium to achieve the desired texture for nono, in combination with the best indigenous isolate which exhibited the desired sensory characteristics of nono (*L. rhamnosus*). *Lactobacillus rhamnosus* has not been previously reported by authors as a part of the nono microflora, which makes it one of the key findings in this study.

The final starter culture consortium used for the fermentation of nono was a 1% combination of *L. rhamnosus*, *L. plantarum*, *L. casei*, and *L. lactis*; this consortium yielded a product very similar to the popularly consumed commercial product, both in terms of physical and sensory properties. Comparing the nono fermented with this starter culture to the commercially purchased nono used as a control, there was a significant (*p* < 0.05) difference in the pH, total titratable acidity, viscosity, and a* and b* dairy colors. The starter culture consortium had a significantly lower pH (4.471), which varies from the results of [12], who reported a pH range of 5.5–7.9. The low pH seen in this study is expected because *Lactococcus lactis* and *Lactobacillus casei* are known acidifiers in milk fermentation [20]. The starter culture consortium exhibited a mean total titratable acidity of 5.50 g/L, which differs from the findings of both Abdulrahman [1] and Omola [3]. This could be further explained as a possible result of differences in the combined organic acid production from each member of the lactic acid starter culture consortium. On the other hand, the commercial nono had a significantly higher viscosity (168 mpa.s) than that of the starter culture consortium (107.5 mpa.s). The possible reason behind this finding is related to the long hours of milk boiling before fermentation in commercial nono production, which possibly result in the loss of volume, coupled with the action of indigenous starter cultures that bind water through exopolysaccharide formation. Comparatively, the milk used for fermentation in this study was not boiled, but it was ultra-high-temperature (UHT)-pasteurized milk; thus, the difference in the heat treatment of both fermentation substrates possibly constituted the basic difference in their texture and viscosity. Lactalbumin denaturation and casein precipitation usually occur when milk is exposed to heating for a prolonged time, which impacts the gel structure of the milk [24] and could also be an additional reason for the difference in viscosity seen in both nono samples. However, the notable similarity in the viscosity of both the commercial nono and the test nono can be attributed to Bingham plastic behavior. It was observed that the viscosity value obtained from the starter culture consortium partly corresponds with the findings of Omola et al. [3], while the value of the commercial nono varied from it considerably.

Both products also had a notable difference in color, with the commercial product having a darker brown color than the starter culture consortium. This characteristic dark brown color of commercial nono could be a result of lactose caramelization and/or the production of humin and melanin commonly associated with protein breakdown in the presence of sugars at high temperatures [26]. This study used the CIE L*a*b* color system to calculate the lightness, redness, and yellowness, respectively, of both commercial nono and that made from the starter culture consortium. There was no significant difference in the lightness (L*) and redness (a*) of both products, but there was a significant difference in their yellowness (b*) (*p* = 0.0017), with the commercial nono having much higher b* value. This could possibly be a result of the long hours of boiling/caramelization it may have gone through before fermentation, coupled with the possible actions of some vitamins like riboflavin [27]. The change in color (∆E) between both samples was 6.71, which implies that most people could easily tell the difference in color between the products. To our knowledge, no previous study has examined the chromatic components of nono, which is also another key finding in this study, particularly given that Milovanovic [28] opined that appearance attributes, like color, are very important in milk products because they often influence consumers’ choices.

Sensory evaluation showed that nono made from the select starter culture consortium had an overall acceptance of 7.65 ± 0.47 on a nine-point hedonic scale. However, the untrained panelists chose the commercial nono over the product made from the constituted starter culture on the basis of taste, aroma and texture. It is assumed that the major difference between the experimentally fermented product and that available on the market stems from the nature of the starting material used for fermentation. This study used UHT-pasteurized milk, while the nono consumed in Nigeria is made from milk boiled for 10–12 h; this boiling is presumed to impact the color, aroma, and texture of the milk prior to fermentation. The sensory evaluation result obtained in this study partly corresponds with that of [12], who reported an overall acceptance of 7.01 ± 0.02. A key difference between this starter culture study and [12] is that the latter used isolates individually but did not proceed to check their fermentation capacities as a co-culture consortium, which was done in this study.

## 5. Conclusions

Synergistic interactions among lactic acid bacteria are required to produce nono with the desired characteristics. Starter culture formulation for nono provides a standardized product with known consistency and is beneficial for solving food safety problems caused by improper food sanitation processes. It ushers in an era of wholesome product formulation for the milkmaids who earn a living from this craft, as well as providing extra opportunity for people outside of Nigeria and West Africa to be able to make nono in their homes using the starter cultures. This study provides a conduit for introducing nono to the rest of the world, just like yoghurt, cheese, dahi, and kefir.

## Figures and Tables

**Table 1:** Identification of indigenous isolates from nono.

Isolate Groups	Colony Morphology		Biochemical Tests		Presumptive Organisms	16s rDNA Identity
		Gram stain	Catalase	Oxidase		
A	Glistening, punctiform whitish colonies with entire margins and smooth appearance	Positive rods	Negative	Negative	*Lactobacillus* sp.	*L. fermentum*
B	Punctiform milkish colonies with entire margins and smooth appearance.	Positive rods	Negative	Negative	*Lactobacillus* sp.	*L. paracasei*
C	Circular milkish colonies with glistening appearance	Positive cocco-bacilli	Negative	Negative	*Lactobacillus* sp.	*L. fermentum*
D	Circular whitish colonies with slimy appearance	Positive rods	Negative	Negative	*Lactobacillus* sp.	*L. fermentum*
E	Punctiform, milkish colonies with slimy appearance	Positive cocco-bacilli	Negative	Negative	*Lactobacillus* sp.	*L. rhamnosus*

**Table 2:** Preliminary screening of the sensory properties of nono made from individual isolates.

Isolates	Sensory Property of Fermented Product
*Lactobacillus fermentum* 1	Nono aroma, fresh milk taste, and white color
*Lactobacillus paracasei*	Nono taste only
*Lactobacillus fermentum* 2	Fresh milk taste only
*Lactobacillus fermentum* 3	Nono aroma, fresh milk taste, and white color
*Lactobacillus rhamnosus*	Nono appearance, taste, and aroma
*Lactobacillus lactis*	Yogurt aroma and taste
*Lactobacillus plantarum*	Fresh milk taste and yogurt aroma
*Lactobacillus casei*	Fresh milk taste and yogurt aroma

**Table 3:** Evaluation of the effects of the best starter culture consortium on the physical properties of nono.

Samples	pH	Moisture Content (%)	Water Activity	Viscosity (mpa.s)	Total Titratable Acidity		Color	
						L*	a*	b*
Fresh milk	6.66 ± 0.02 ^b^	87.12 ± 0.01 ^a^	0.99 ± 0.00 ^a^	5.33 ± 0.29 ^a^	3.50 ± 0.20 ^a^	79.52 ± 1.58 ^a^	−1.99 ± 0.05 a	−0.38 ± 0.63 ^a^
Commercial nono	6.56 ± 0.05 ^b^	87.51 ± 0.25 ^a^	0.99 ± 0.00 ^a^	165.3 ± 2.52 ^c^	2.93 ± 0.12 ^a^	85.59 ± 1.45 ^a^	−3.19 ± 0.09 ^b^	4.09 ± 0.28 ^c^
Starter culture mix	4.49 ± 0.02 ^a^	87.30 ± 0.02 ^a^	1.00 ± 0.01 ^a^	113.7 ± 7.64 ^b^	5.50 ± 0.30 ^b^	83.45 ± 2.52 ^a^	−3.02 ± 0.16 ^b^	2.73 ± 0.44 ^b^
*p*-value	0.002	0.144	0.066	0.003	0.003	0.104	0.007	0.0017

Mean values in the same column with different letters are significantly different (*p* ≤ 0.05).

**Table 4:** Sensory analyses of test nono samples made from the starter culture mix.

Attributes	Mean ± S.D	*p*-Value
Appearance	8.04 ± 0.70	0.584
Taste	8.37 ± 0.69	0.343
Aroma	7.49 ± 0.56	0.145
Texture	7.05 ± 0.89	0.015
Overall acceptance	7.65 ± 0.47	0.009

## Data Availability

The raw data supporting the conclusions of this article will be made available by the authors on reasonable request.
